# Editorial: Diversity in Medical Education

**DOI:** 10.15694/mep.2018.000001.1

**Published:** 2018-01-16

**Authors:** Petra Verdonk, Janusz Janczukowicz

**Affiliations:** 1 Vrije Universiteit- Amsterdam; 2 Medical University of Lodz

**Keywords:** diversity, gender

## Abstract

This article was migrated. The article was marked as recommended.

While medical schools need to address the health care needs of diverse populations and to accommodate to the diverse needs of their students and staff, addressing and studying diversity in medical education remains challenging. In teaching cultural competence, several approaches are identified, including a cultural expertise approach, a cultural sensibility approach and a cross-cultural approach with the more recently emerging call for reflexivity. We propose the analysis of diversity related issues at three distinct levels: fixing the numbers, fixing the institutions and fixing the knowledge.

## Introduction

Medical schools need to address the health care needs of diverse populations (1, 2), and to accommodate to the diverse needs of their own students and staff (3,4). Despite the advocacy for cultural competence already for decades, and despite the influx of female and minority students, the uptake of diversity issues in all facets of medical education has been slow. Issues are for instance addressing the (under)representation of black and minority students (5), transforming learning climate and organizational cultures (6,7), and integrating diversity issues in curricula (2,6-9).

In teaching cultural competence, several approaches are identified (2,10): (1) a cultural expertise approach which is grounded in biomedicine and fact-driven; (2) a cultural sensibility approach which is grounded in social constructivism and aims to increase awareness of sociocultural aspects of patient-provider encounters, and; (3) a cross-cultural approach which rather focusses on the development of communication skills and tools to improve the patient-provider interaction (10). More recently a call for reflexivity has also emerged, which addresses the need for a proper understanding of future doctors’ and teachers’ own social identities and position from an intersectional perspective, and how their identities affect knowledge, interactions and decision-making in health care and medical education (11,12). Most likely, different approaches to education establish different outcomes in future doctors, but research is scarce (1).

Addressing and studying diversity in medical education remains challenging. Despite the interest in change and the ‘business case’ ideal of diversity as being synonymous with ‘innovation’ and ‘quality’ in health care and education, in practice addressing diversity remains painful and ambiguous. For instance, university mission statements promote diversity and excellence and innovation while half-heartedly implementing its consequences, rather ‘doing the documents’ than doing the transformation (13). Furthermore, addressing diversity sometimes reinforces stereotypes rather than targets them (2), while discussing diversity may evoke emotive responses in classrooms rather than create ‘positive interactions’ between students and staff (14,15). For these and other obvious reasons, shying away from diversity’s difficulties can be understandable. However, renewed interest in diversity issues is fueled by mass immigration and political developments such as rising nationalism and refugee crises.

Recently, AMEE conferences in Glasgow (2015), Barcelona (2016), and Helsinki (2017) have shown that medical educators are interested in diversity issues. In 2017, a range of abstracts from across the world issued the integration of sex and gender issues in Germany, sexual and gender minority health issues in the Netherlands, the UK, and Turkey, teaching cultural competence in future doctors in Hong Kong, Taiwan, South-Africa, and Romania, or the experiences of female, minority and indigenous students in medical education in Slowakia, Australia, Pakistan, and Sweden.

Thus, from an understanding that addressing diversity is first and foremost a social justice issue (13) and thus, a basic tenet of quality of education and health care, we are more than happy to welcome this special issue. We encourage the submission of articles from a diversity of authors across the globe including medical students, about a range of ‘different differences’ and their intersections, and from many different angles. For the sake of structure and organization, we propose an analysis at three distinct levels (16) described below.

## Fixing the numbers

*Fixing the numbers* refers to the composition of the student body in medical schools or to the numbers of female and minority students and staff in education, teaching, and research, thus, to ‘body counts’ and equal opportunity. Compositional diversity aims for an adequate reflection of the population within the composition of student, teaching staff and hospital staff demographics (15). Yet, fixing the numbers is easier said than done. Although female students outnumber male students in medical schools, women are still underrepresented at higher levels in the medical profession (17). Gendered processes of inclusion and exclusion still play a role in medical training and more so at the expense of female graduates (3). Likewise, cultural background, ethnicity, or ‘race’ (despite its ban from EU regulations ‘race’ is still a social construct also in European societies) are still grounds for in- and exclusion (4) as are other diversity aspects such as disability or sexual diversity. This brings us to the second level.

## Fixing the institutions

*Fixing the institutions* refers to learning climates and organizational culture of medical schools: to legal regulations and professional standards, for instance whether admission policies are dedicated to equality, but also to unspoken assumptions and values behind ways of doing and behind claims of neutrality and objectivity. Medical schools often aim to target exclusion and oppression such as sexual harassment, heterosexism, and racism, and struggle to transform university cultures and lift biases (18). Ultimately, schools strive for interactional diversity with positive interactions between students and staff with diverse background (14). But bringing students together is insufficient to establish positive interactions - all too often, students are segregated due to processes of Othering (4,13) and positive interactions can only occur when we increase our competences to deal with ‘emotive’ issues (13,19). And when minoritized, underrepresented, or subordinate groups are supposed to fully accommodate to the norms of dominant groups without reflection (11,19), new learning climates in ‘transformed institutions’ are unlikely to emerge. This brings us to the third level.

## Fixing the knowledge

*Fixing the knowledge* emphasizes the consequences of exclusion for our knowledge base. At this level, researchers and educators focus on curricular and knowledge content, on analyzing how a diversity perspective enhances knowledge, and thus, health care practices. Fixing the knowledge, both from a biomedical and constructivist perspective, may be promising not only to improve future doctors’ competences, but also because it creates spaces for intentionally discussing identities and what they mean for health, for health care, and thus, for teaching and learning (11,12,19). For that, some soul searching is required. We must uphold basic human rights to safeguard and increase access and quality of education and care for everyone. Given that exclusion and injustice as a problem are not going home any time soon, neither of us can go home (20). Social justice, and thus, actively thinking about dealing with diversity, needs us now more than ever. We look forward to wide participation in this discussion.

## Notes On Contributors

Dr. Petra Verdonk (p.verdonk@vumc.nl) is an occupational health psychologist with a PhD in integrating gender perspective in medical curricula. She is an assistant professor at the department of Medical Humanities at VUMC Amsterdam. Her main areas of expertise and research interest include gender and intersectionality/ diversity issues in medical education and public health.

Dr. Janusz Janczukowicz MD, MMEd, PhD (janusz.janczukowicz@umed.lodz.pl) is a head of Centre for Medical Education at the Medical University of Lodz. He is also the Member of the AMEE Executive Committee and the Board Member of the European Institute of Women’s’ Health, with main expertise in professionalism, diversity, social competence and general medical education.

## Declarations

Dr. Petra Verdonk and Dr. Janusz Janczukowicz are guest Theme Editors for AMEE MedEdPublish for the theme of Diversity in Medical Education.
