# Supplementary material for: Editorial: Diversity in Medical Education
Source: MedEdPublish (2016). 2018 Jan 16;7:1. [Version 1] doi: 10.15694/mep.2018.000001.1 (PMC10704425; doi:10.15694/mep.2018.000001.1)
Supplement: Peer review history

# Reviewer report: Reviewer response for version 1

**DOI:** 10.21956/mep.19494.r28804

**Published:** 2018-04-13

**Authors:** Barbara Jennings

**Affiliations:** 1 Norwich Medical School

This review has been migrated. The reviewer awarded 5 stars out of 5 This is a really valuable themed issue of the journal and the opening editorial presents a helpful framework for the discussion of diversity in medical education.The editors have also provided us with a thorough and accessible overview of the relevant literature. This is an interesting and engaging paper with an invitation to engage in scholarship and research about how diversity is, and/or should be, included in the medical curriculum.

Reviewer Expertise:

NA

No decision status is available

# Reviewer report: Reviewer response for version 1

**DOI:** 10.21956/mep.19494.r28803

**Published:** 2018-01-18

**Authors:** Trevor Gibbs

**Affiliations:** 1 AMEE

This review has been migrated. The reviewer awarded 5 stars out of 5 An excellent editorial introduction to a very important issue, which the authors quiet rightly point out is never going to go away; indeed it is only going to grow in importance. I like the three- pronged way the authors have highlighted the way that the issue can be analysed and I look forward to reading the many papers that I hope this editorial attracts

Reviewer Expertise:

NA

No decision status is available

# Reviewer report: Reviewer response for version 1

**DOI:** 10.21956/mep.19494.r28802

**Published:** 2018-01-16

**Authors:** Subha Ramani

**Affiliations:** 1 Harvard Medical School, Brigham and Women's Hospital

This review has been migrated. The reviewer awarded 5 stars out of 5 I commend the theme editors for their work and emphasizing this important area of health professions education. Given that this is an editorial and a prelude to more detailed articles, I believe they have done a very good job of introducing the theme and outlining strategies for educational institutions to apply. The approach using the 3 areas to fix was useful to me in conceptualizing inclusion and diversity in education.

Reviewer Expertise:

NA

No decision status is available

# Reviewer report: Reviewer response for version 1

**DOI:** 10.21956/mep.19494.r28805

**Published:** 2018-01-16

**Authors:** Rashmi Kusurkar

**Affiliations:** 1 Amsterdam UMC, Faculty of Medicine Vrije University Amsterdam

This review has been migrated. The reviewer awarded 4 stars out of 5 I agree with the perspective put forth in this editorial that dealing with diversity in medical education involves fixing the numbers, fixing the institutions, and fixing the knowledge. I think what would add to this approach is involving patients in providing the diversity perspective while designing educational courses and modules. I look forward to the contributions made by my fellow medical educators to this special issue.

Reviewer Expertise:

NA

No decision status is available

# Reviewer report: Reviewer response for version 1

**DOI:** 10.21956/mep.19494.r28806

**Published:** 2018-01-16

**Authors:** Julie Hunt

**Affiliations:** 1 Lincoln Memorial University

This review has been migrated. The reviewer awarded 4 stars out of 5 This commentary on the state of diversity in medical education is timely and relevant. The authors highlight three areas for growth: fixing the numbers, the institutions, and the knowledge. The authors do a good job of referencing relevant social; constructs. One of the strengths of this commentary is its brevity, though at the same time, I am left wanting a more thorough exploration of each of the "fixing" areas.

Reviewer Expertise:

NA

No decision status is available

# Reviewer report: Reviewer response for version 1

**DOI:** 10.21956/mep.19494.r28801

**Published:** 2018-01-16

**Authors:** P Ravi Shankar

**Affiliations:** 1 American International Medical University

This review has been migrated. The reviewer awarded 4 stars out of 5 I have long been interested in ‘Diversity’ in health professions education and look forward to this theme issue. Many groups are under-represented in medical schools while affirmative action and reservations have fixed a certain amount of seats for various groups. Offshore Caribbean medical schools mainly cater to students from the United States and Canada. In my previous school, Xavier University School of Medicine in Aruba the majority of the students was second generation Americans or Canadians of Asian ancestry. At my present school a large proportion of the students are Indian or Nigerian citizens. Accreditation agencies are increasingly interested in knowing about measures the school is taking to strengthen diversity among both students and faculty. On small Caribbean islands female faculty are lesser in number and many join as a couple with their husbands or partners. The ethnicity and background of the group owning the school does influence the geographical origin of faculty members. Standards of remuneration and working conditions vary widely among Caribbean medical school also influencing the faculty composition. Also students will be doing their clinical rotations and practice in various locations hence educating them about cultural competence and dealing with a diverse patient population is also important.

Reviewer Expertise:

NA

No decision status is available
